# Neurofibroma of the Posterior Ankle: A Case Report

**DOI:** 10.7759/cureus.94409

**Published:** 2025-10-12

**Authors:** Kimia Targhi, Melodie Mope

**Affiliations:** 1 College of Medicine, Florida State University, Tallahassee, USA; 2 Department of Family Medicine, Florida State University, Tallahassee, USA

**Keywords:** foot mass, peripheral nerve sheath tumors, soft tissue tumor, solitary neurofibroma, subcutaneous mass

## Abstract

Neurofibromas are benign peripheral nerve sheath tumors that most commonly occur on the trunk, head, and neck. In the foot and ankle, they are uncommon, and those arising from the posterior heel are exceedingly rare, representing an unusual site of occurrence. We report the case of a 49-year-old woman with a persistent lesion on the posterior aspect of her right heel, initially misdiagnosed as a callus due to its superficial appearance and resemblance to common mechanical lesions. Physical examination revealed a firm, mobile, tan-colored mass. The lesion was small and appeared benign so imaging was not performed prior to excision. Histopathological analysis revealed spindle-shaped cells within a loose collagenous stroma consistent with a benign neurofibroma. The patient had complete resolution of symptoms after excision and no recurrence at the six-month follow-up. This case illustrates how neurofibromas in uncommon locations such as the posterior heel can mimic more common conditions, delaying accurate diagnosis. Persistent or atypical heel lesions unresponsive to conservative therapy should prompt early biopsy and histopathological evaluation to ensure timely and appropriate management.

## Introduction

Neurofibromas are benign, slow-growing tumors originating from peripheral nerves, composed of Schwann cells, fibroblasts, and perineural cells. Although commonly associated with neurofibromatosis type 1 (NF1) and type 2 (NF2), approximately 90% occur sporadically, without any underlying syndromic association [1].

Localized neurofibromas can arise anywhere on the body, most often on the trunk, head, and neck. In the foot and ankle, however, they represent only 5.4% of benign soft tissue tumors [2]. Within this subset, lesions of the posterior heel or adjacent to the Achilles tendon are exceedingly rare and, to our knowledge, have not been documented in the literature to date. Because of their rarity in this anatomic region, such lesions are often overlooked or mistaken for more common mechanical and inflammatory conditions.

Clinically, heel masses are far more likely to represent calluses, bursitis, lipomas, or epidermoid cysts, and as a result, neurofibromas in this location may remain undiagnosed for years. This diagnostic overlap can lead to repeated conservative management attempts, delayed biopsy, and prolonged patient discomfort. For non-specialist clinicians, recognizing when a chronic or atypical "callus" fails to behave as expected is crucial, as early histopathological evaluation can prevent misdiagnosis and unnecessary treatment. 

This report presents a rare case of a posterior heel neurofibroma initially misdiagnosed and treated as a callus for several years. It underscores both the diagnostic pitfalls associated with heel lesions and the importance of considering soft tissue neoplasms in the differential diagnosis when conservative therapy fails. By expanding awareness of this uncommon presentation, this case aims to guide timely recognition and improve patient outcomes through early biopsy and accurate diagnosis.

## Case presentation

A 49-year-old woman presented with a complaint of a persistent callus on the posterior aspect of her right heel. She reported that the lesion had been present for approximately five years and was previously diagnosed and treated as a callus by a dermatologist without improvement. Over time, it gradually increased in size and began to cause discomfort when wearing closed-back shoes, prompting her to seek further evaluation.

**Figure 1:**
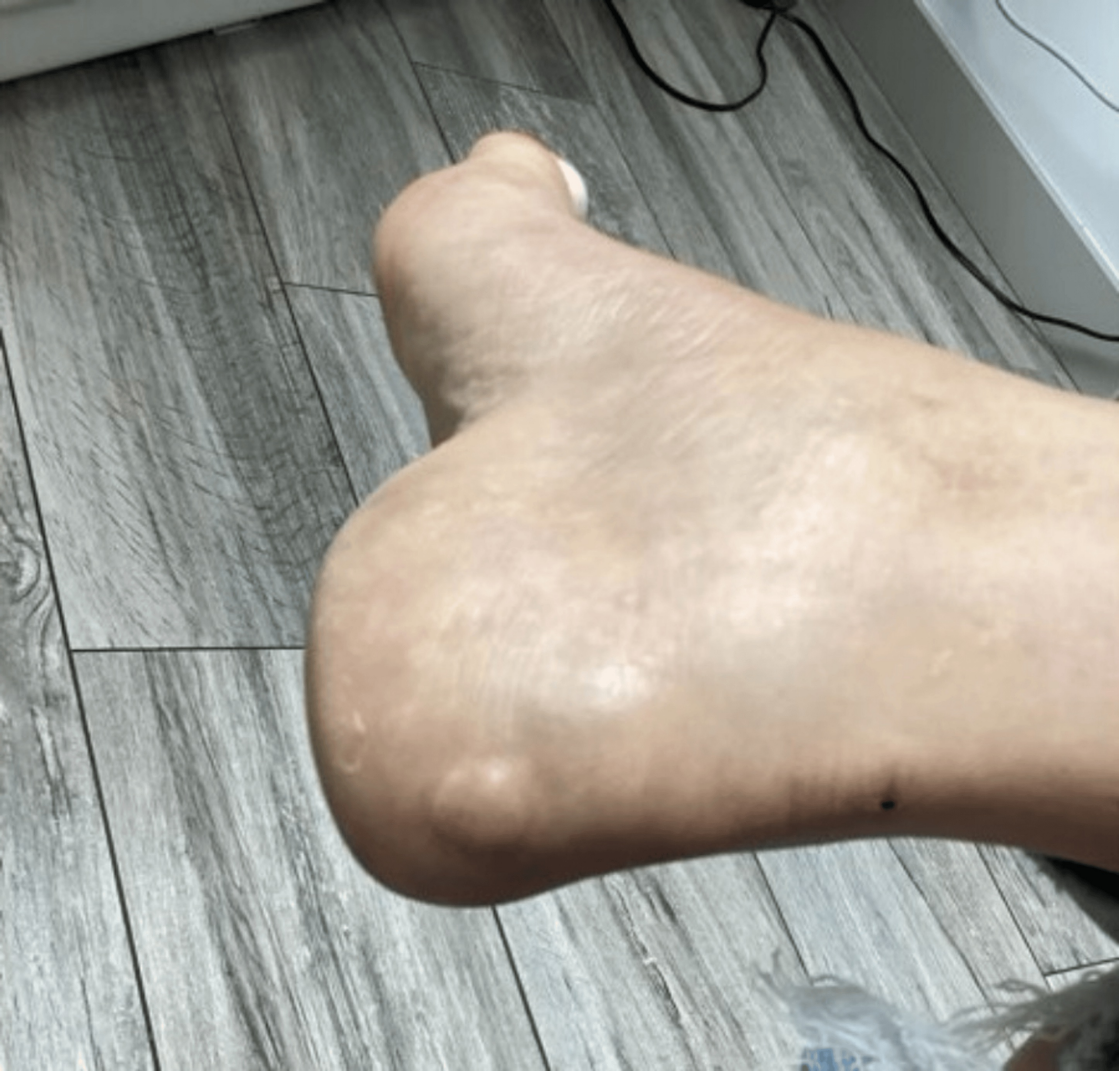
A 1.5 × 1.5 × 0.5 cm well-circumscribed, tan-colored soft tissue mass over the posterior right heel.

On physical examination, the lesion appeared as a 1.5 × 1.5 × 0.5 cm well-circumscribed, tan-colored soft tissue mass over the posterior right heel (Figure 1). It was firm, mildly compressible, and mobile within the subcutaneous tissue. The lesion was non-tender at rest but elicited mild discomfort on palpation. The overlying skin was intact, with no ulceration, drainage, or discoloration. A systemic examination revealed no café-au-lait macules, cutaneous neurofibromas, axillary/inguinal freckling, Lisch nodules, or other stigmata of NF1 or NF2.

Given the lesion's chronicity, superficial location, and benign clinical appearance, it was suspected to be an epidermoid cyst. Imaging with ultrasound or MRI was not pursued because the lesion was small, superficial, and amenable to excision and the patient preferred direct removal. The mass was excised in its entirety and sent for histopathological examination.

**Figure 2:**
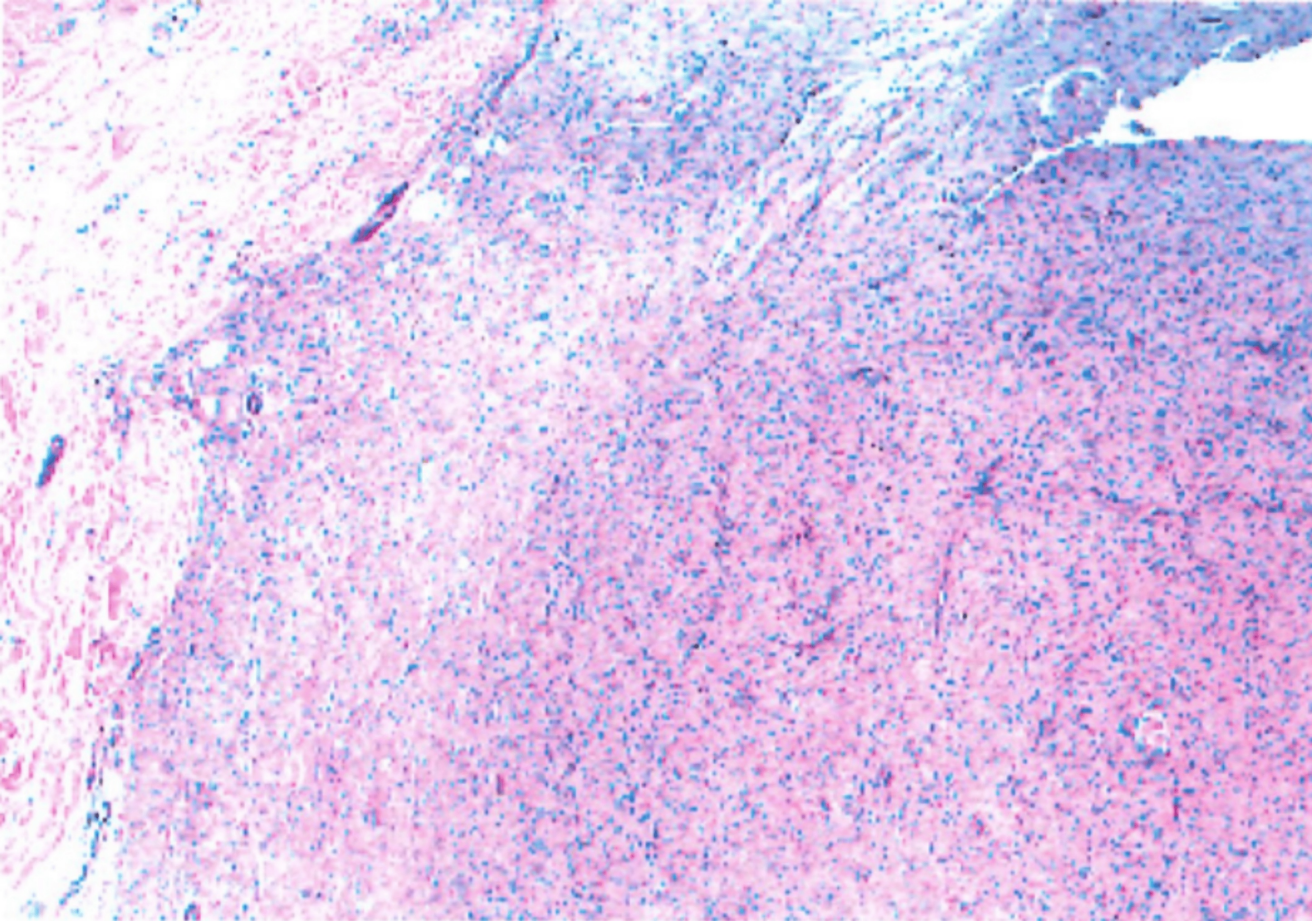
The tissue demonstrates a normal-appearing epidermis overlying a dermal nodule composed of spindle-shaped cells in a loose fibrous stroma.

Histologic evaluation revealed a normal-appearing epidermis overlying a dermal nodule composed of spindle-shaped cells in a loose fibrous stroma, consistent with a benign neurofibroma (Figure 2). 

**Figure 3:**
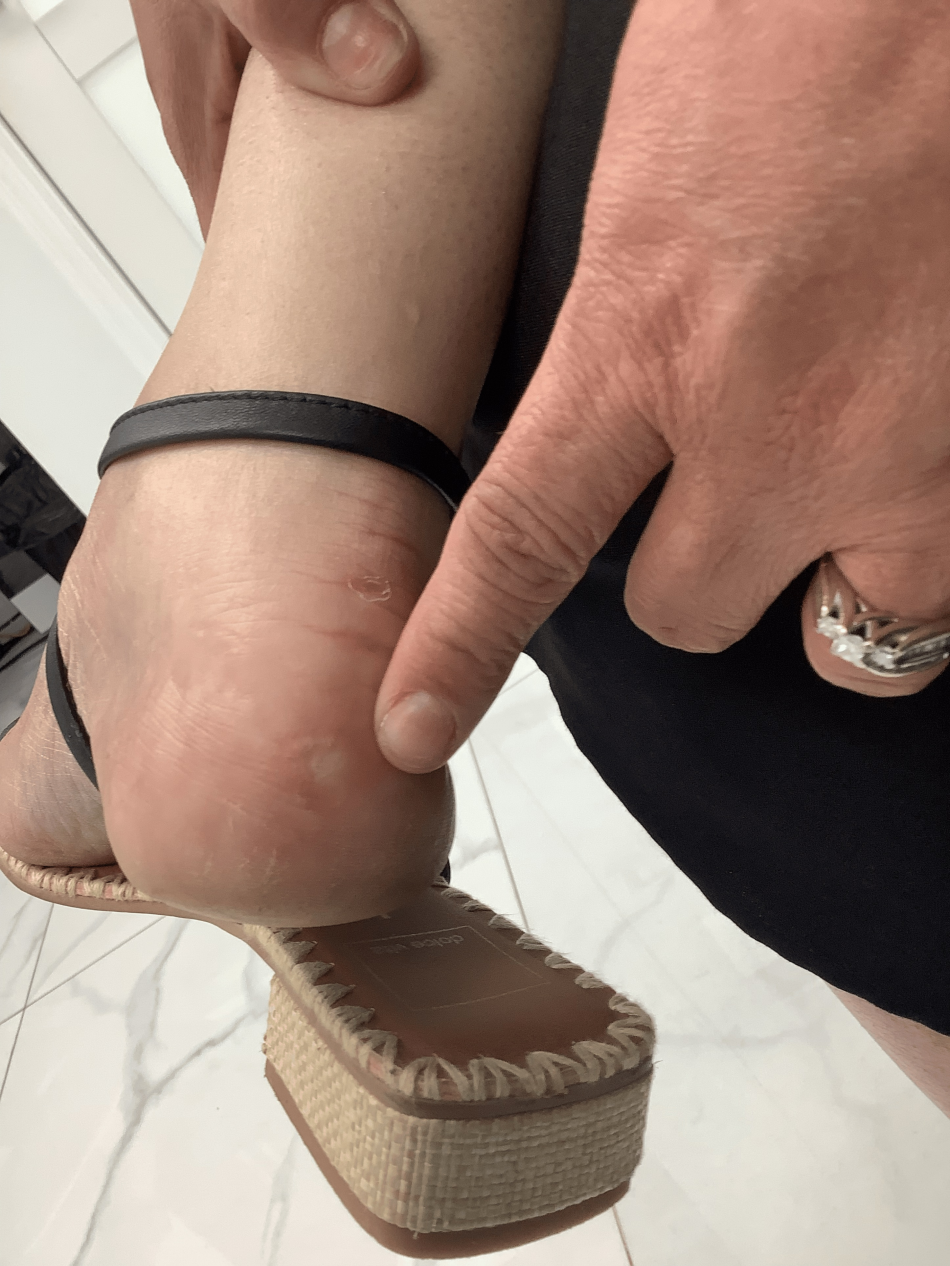
Posterior aspect of the right heel one year after excision, demonstrating complete healing and no evidence of recurrence.

The patient was seen one month after excision and again one year later for an unrelated foot condition. During both visits, she reported complete resolution of her prior symptoms with no clinical evidence of recurrence (Figure 3). 

## Discussion

Neurofibromas are benign peripheral nerve sheath tumors that typically present as soft, slow-growing nodules. In both sporadic and syndromic cases, they are associated with deletions or mutations in the NF1 gene [1]. These tumors most commonly occur on the trunk, head, and neck and are relatively uncommon in the foot and ankle, where they account for only 5.4% of benign soft tissue tumors [2]. Given the scarcity of reported cases, neurofibromas of the posterior heel are particularly rare, making diagnosis challenging when they present in this location. 

The lesion in this patient appeared as a firm, superficial nodule over the posterior calcaneus, which initially contributed to its misdiagnosis. In this anatomic region, more common conditions such as calluses, bursitis, epidermoid cysts, fibromas, ganglion cysts, lipomas, or dermatofibromas are usually considered first. These overlapping clinical features underscore the importance of maintaining a broad differential diagnosis when evaluating heel masses.

Potential etiologic factors for solitary neurofibromas include repetitive mechanical irritation or prior trauma, as described in previous reports. For example, one case report described a 23-year-old man with a 4 × 3 cm lump in the dorsolateral forefoot that first appeared a few weeks after a fall sustained during military training. The lesion progressively enlarged over three years, and histopathology ultimately confirmed the diagnosis of diffuse neurofibroma [3]. Solitary sporadic cases have also been described, including a 17-year-old girl with a gradually enlarging 6 × 3 cm swelling in the distal third of the right leg, for which excisional biopsy established the diagnosis of solitary neurofibroma [4]. Genetic predispositions have been established in syndromic cases, but no environmental triggers or inherited factors were evident in this patient, who lacked the stigmata of NF1 or NF2.

Radiological imaging can assist in the evaluation of atypical soft tissue masses. Ultrasound is often employed as a first-line modality given its high axial resolution and ability to differentiate solid from cystic lesions. In contrast, MRI is considered the gold standard for peripheral nerve sheath tumors. It provides superior soft tissue characterization and delineation of lesion boundaries, which can aid in surgical planning and in distinguishing neurogenic from non-neurogenic tumors [5]. One of the limitations of our case report is the absence of pre-excision imaging. Because the lesion was small, superficial, and clinically benign in appearance, imaging was deferred, and excision was pursued directly. However, MRI could have provided additional diagnostic information and may have demonstrated the lesion's neurogenic origin more clearly.

Histologically, neurofibromas consist of spindle-shaped Schwann cells with poorly defined cytoplasmic borders, embedded in a loose myxoid to collagenous stroma. Other histologic features include small, hyperchromatic nuclei and absent to minimal mitoses. Immunohistochemically, neurofibromas demonstrate variable but often diffuse S100 positivity in Schwann cells and CD34 expression in fibroblastic elements [6]. Epithelial membrane antigen (EMA) stain is generally negative except in plexiform neurofibromas, in which EMA stains the surrounding perineurium of the nerve bundle [7]. Another limitation of this case report was that immunohistochemical stains such as S100 and CD34 were not performed. However, the lesion's classic spindle cell morphology and clinical context were considered diagnostic. 

Although no gold-standard treatment exists for neurofibromas, physical removal remains the cornerstone of management. This can be achieved through surgical excision with primary closure, modified biopsy excision, or destructive techniques such as CO₂ laser, electrodessication, and ablation. Limitations of physical removal include tumor regrowth from incomplete excision, significant scarring, and substantial financial costs. The latter remains a major barrier as most insurance companies classify neurofibroma removal as a cosmetic rather than medically necessary procedure. Moreover, while these interventions address existing lesions, they do not prevent the development of new neurofibromas [8]. Solitary neurofibromas do not carry a risk of malignant transformation [9], unlike plexiform neurofibromas, which develop in over 30% of patients with NF1 and are associated with a poor five-year survival rate [10]. After one year, our patient remained asymptomatic with no evidence of recurrence. This favorable outcome reinforces that complete excision is typically curative for solitary, sporadic neurofibromas.

This case contributes to the limited literature on neurofibromas of the posterior heel and illustrates the importance of maintaining a broad differential diagnosis when evaluating soft tissue lesions of the foot, particularly those that persist, evolve, or fail to respond to conservative treatment. It also emphasizes biopsy as a critical diagnostic tool when the clinical presentation and expected outcomes do not align.

## Conclusions

This case highlights how uncommon lesion locations, such as the posterior heel, can obscure the diagnosis of otherwise well-characterized tumors like neurofibromas. When common treatments fail or a lesion deviates from expected behavior, biopsy should be pursued as histopathological evaluation remains the most reliable method for establishing an accurate diagnosis. Recognizing atypical presentations not only ensures timely treatment but may also prompt clinicians to reconsider routine assumptions in foot and ankle pathology.

## References

[1] Messersmith L, Krauland K (2023). Neurofibroma. StatPearls.

[2] Hughes P, Miranda R, Doyle AJ (2019). MRI imaging of soft tissue tumours of the foot and ankle. Insights Imaging.

[3] Nasser AA, Al-Saad S, Awad RK, Alkhalifa F (2018). Post traumatic diffuse neurofibroma in the foot: an unusual presentation. Open Orthop J.

[4] Kalyanpur A, Dsouza R, Kurien B (2023). A case of solitary neurofibroma involving the lower limb in a young tribal girl. Curr Med Issues.

[5] Shirodkar K, Hussein M, Reddy PS (2025). Imaging of peripheral intraneural tumors: a comprehensive review for radiologists. Cancers (Basel).

[6] Nagrani NS, Bhawan J (2022). Histopathological variants of cutaneous neurofibroma: a compendious review. Dermatopathology (Basel).

[7] Theaker JM, Gatter KC, Puddle J (1988). Epithelial membrane antigen expression by the perineurium of peripheral nerve and in peripheral nerve tumours. Histopathology.

[8] Chamseddin BH, Le LQ (2020). Management of cutaneous neurofibroma: current therapy and future directions. Neurooncol Adv.

[9] Ortonne N, Wolkenstein P, Blakeley JO (2018). Cutaneous neurofibromas: current clinical and pathologic issues. Neurology.

[10] Evans DG, Baser ME, McGaughran J, Sharif S, Howard E, Moran A (2002). Malignant peripheral nerve sheath tumours in neurofibromatosis 1. J Med Genet.

